# Availability of comprehensive emergency obstetric and neonatal care in developing regions in Ethiopia: lessons learned from the USAID transform health activity

**DOI:** 10.1186/s12913-022-08712-w

**Published:** 2022-11-02

**Authors:** Gugsa Nemera Germossa, Tamiru Wondie, Mulusew Gerbaba, Eyob Mohammed, Wondwossen A. Alemayehu, Asayehegn Tekeste, Eden Ahmed Mdluli, Thomas Kenyon, Deborah Collison, Sentayehu Tsegaye, Yared Abera, Derebe Tadesse, Wakgari Binu Daga, Tamrat Shaweno, Mohammed Abrar, Ahmed Ibrahim, Mebrie Belete, Salah Esmael, Daniel Tadesse, Yibeltal Kiflie Alemayehu, Girmay Medhin, Mekdes Daba Fayssa

**Affiliations:** 1 School of Nursing, Jimma University, Jimma, Ethiopia; 2 Project HOPE, USAID Transform Health in Developing Regions, Addis Ababa, Ethiopia; 3 Department of Epidemiology, Jimma University, Jimma, Ethiopia; 4 Ethiopian Society of Obstetrics and Gynecology, Addis Ababa, Ethiopia; 5 Project HOPE, Washington DC, Washington USA; 6 USAID Transform Health in Developing Regions, Amref Health Africa, Addis Ababa, Ethiopia; 7 School of Public Health, Wolaita Sodo University, Sodo, Ethiopia; 8 Africa Centers for Diseases Control and Prevention (Africa CDC), Addis Ababa, Ethiopia; 9 Amref Health Africa, Afar Regional Office, Semera, Ethiopia; 10 AMref Health Africa, Somali Regional Office, Jijiga, Ethiopia; 11 AMref Health Africa, Gambela Regional Office, Gambela, Ethiopia; 12 Amref Health Africa, Beneshangul Regional Office, Asosa, Ethiopia; 13 MERQ Consultancy PLC, Addis Ababa, Ethiopia; 14 Department of Health Economics, Management, and Policy, Jimma University, Jimma, Ethiopia; 15 Aklilu Lemma Institute of Pathobiology, Addis Ababa University, Addis Ababa, Ethiopia; 16 St. Paul’s Hospital Millennium Medical College, Addis Ababa, Ethiopia

**Keywords:** Emergency obstetric care, Emergency newborn care, Mentoring, developing regions

## Abstract

**Background:**

In collaboration with its partners, the Ethiopian government has been implementing standard Emergency Obstetric and Neonatal Care Services (CEmONC) since 2010. However, limited studies documented the lessons learned from such programs on the availability of CEmONC signal functions. This study investigated the availability of CEmONC signal functions and described lessons learned from Transform Health support in Developing Regional State in Ethiopia.

**Method:**

At baseline, we conducted a cross-sectional study covering 15 public hospitals in four developing regions of Ethiopia (Somali, Afar, Beneshangul Gumz, and Gambella). Then, clinical mentorship was introduced in ten selected hospitals. This was followed by reviewing the clinical mentorship program report implemented in all regions. We used the tool adapted from an Averting Maternal Death and Disability tools to collect data through face-to-face interviews. We also reviewed maternal and neonatal records. We then descriptively analyzed the data and presented the findings using text, tables, and graphs.

**Result:**

At baseline, six out of the 15 hospitals performed all the nine CEmONC signal functions, and one-third of the signal functions were performed in all hospitals. Cesarean Section service was available in eleven hospitals, while blood transfusion was available in ten hospitals. The least performed signal functions were blood transfusion, Cesarean Section, manual removal of placenta, removal of retained product of conceptus, and parenteral anticonvulsants. After implementing the clinical mentorship program, all CEmONC signal functions were available in all hospitals selected for the mentorship program except for Abala Hospital; the number of Cesarean Sections increased by 7.25% at the last quarter of 2021compared to the third quarter of 20,219; and the number of women referred for blood transfusions and further management of obstetric complications decreased by 96.67% at the last quarter of 2021 compared to the third quarter of 20,219. However, the number of women with post-cesarean Section surgical site infection, obstetric complications, facility maternal deaths, neonatal deaths, and stillbirths have not been changed.

**Conclusion:**

The availability of CEmONC signal functions in the supported hospitals did not change the occurrence of maternal death and stillbirth. This indicates the need for investigating underlying and proximal factors that contributed to maternal death and stillbirth in the Developing Regional State of Ethiopia. In addition, there is also the need to assess the quality of the CEmONC services in the supported hospitals, institutionalize reviews, surveillance, and response mechanism for maternal and perinatal or neonatal deaths and near misses.

**Supplementary Information:**

The online version contains supplementary material available at 10.1186/s12913-022-08712-w.

## Introduction

About 94% of all maternal deaths occur in LLMICs and Sub-Saharan African (SSA) countries account for 86% of the global deaths [1]. In this SSA region, deaths during the neonatal period also accounted for 47%. Furthermore, nearly half of deaths occur within the first 24 h of birth, while the first week of life accounts for 75% of neonatal mortality [2]. Ethiopia is no exception; in 2019, about 412 women per 100,000 live births died from preventable pregnancy related complications [3], and neonatal mortality was estimated to be 33 deaths per 1,000 live births [4]. These figures vary significantly amongst Ethiopian regions and Developing Regional States (DRS) have disproportionately large numbers than the country's other regions.

Some of the many preventable causes of maternal mortality include teen pregnancy, lack of access to health services, poor health-seeking behavior, and seasonal mobility [5–9]. From the provider and health system side, inadequacies in infrastructure, missing/non-functioning equipment, a lack of/interrupted supply of essential medicines, and insufficient number and capacity of health care providers are likely to contribute to the slow progress in reducing maternal deaths and neonatal mortality rates [9]. Cognizant of these facts, the Sustainable Development Goals (SDGs) target to lower maternal mortality to less than 70 deaths per 100,000 live births and infant mortality to less than 12 deaths per 1000 live births in 2030 [1] by improving access to quality emergency obstetric and neonatal care (EmONC) services at health facilities [10].

Emergency obstetric and neonatal care (EmONC) signal functions are cost-effective interventions to treat causes of maternal and newborn mortality [10]. Health facilities are classified as CEmONC facilities if they perform Cesarean Section (CS) and blood transfusion services in addition to the seven Basic EmONC signal functions; namely administering parenteral antibiotics, parenteral uterotonic, parenteral anticonvulsants, manual removal of placenta, removal of retained conceptus products, performing assisted vaginal delivery, and performing basic newborn resuscitation [11].

Considerable evidence from LLMICs suggests that the availability and quality of CEmONC services are among the known high-impact interventions for reducing maternal and neonatal mortality. For instance, if addressed timely, EmONC treats 70–80% of direct obstetric problems [12]. The recent evidence in Ethiopia also showed that access to quality EmONC signal functions prevents 50% to 70% of maternal deaths and reduces neonatal mortality by 10% to 15% [13]. However, facility-based cross-sectional studies [14–18] show variations in availability and performance in CEmONC signal functions. For example, only two out of 43 health facilities in Cameroon [17] and 17.4% of health care facilities in Tanzania [18] provide CEmONC services with all nine signal functions. In the Dire Dawa City Administration in Ethiopia, there is significant heterogeneity in access to CEmONC and the performance of the nine signal functions [14]. To address this, the government of Ethiopia and development partners such as USAID Transform Health in Developing Region (HDR) has been supporting hospitals in DRS to improve access, availability, and quality of CEmONC services [19]. However, the extent of availability of CEmONC signal functions and lessons learned from such supports were not thoroughly documented. Hence, this study investigated the availability of CEmONC services in hospitals in the four DRS in Ethiopia and documented lessons learned from USAID Transform health project interventions.

## Methods

### The study contexts

Ethiopia has 11 regional states and two city administrations. All the regions are not equally developed, and there is observed disparity in educational facilities, health service availability, and important infrastructure, including roads, electricity, and clean water [19]. This study is conducted in poorly developed regions where a consortium of partners led by Amref health Africa has been implementing USAID transform health Activity over the last four years. The overall goal of the Transform HDR is to reduce morbidity and mortality among mothers and under-five children by improving the utilization of quality, high-impact MNCH/FP services in the DRSs in Ethiopia as stipulated in the Health Sector Transformation Plan (HSTP I -2016 – 2020). One of the priority focus areas was increasing access to integrated quality high, impact MNCH/FP services at the health facility through availing comprehensive obstetric care (CEmONC) service at the selected hospitals in Afar, Benishangul-Gumuz, Gambella, and Somali Regional States to help increase the number of healthy mothers who have successful birth outcomes.

Transform Health in Developing Regions activity is implemented in 60 woredas to benefit four million people by supporting more than 1,168 health facilities, including 984 health posts, 169 health centers, and 17 hospitals [19]. The current study was conducted in 15 hospitals across the four DRS in Ethiopia, where pastoralists and agro-pastoralists predominate: Somali, Afar, Benishangul Gumuz, and Gambella. Somali and Afar regions are located in eastern Ethiopia, whereas Benishangul and Gambella regions are located in western Ethiopia.

### Study design

A cross-sectional study was conducted on the availability of CEmONC services from January 1 to February 28, 2019, and lessons learned following the Transform HDR programs implemented from August 1, 2019, to December 2020 in the four DRS states were reviewed and documented.

### Sampling

We selected 15 out of 35 public hospitals in the four DRS purposely based on the availability Integrated Emergency Surgical Officers (IESOs) and operation rooms to assess the baseline CEMONC status. Six of the 15 hospitals were from the Somali region, four from the Afar region, three from Benishangul Gumuz, and the remaining two were from the Gambella region. Then ten hospitals that have IESOs, active operating rooms equipped with the appropriate equipment, and provide Caesarian Section (CS) service were identified for the implementation of a clinical mentorship program (CMP) (Supplementary Files 1 and 2).

### Interventions: Brief descriptions of the project

The intervention consists of two interrelated activities: the CEmONC clinical mentorship program and general health facility support.

### Intervention component 1. CEmONC Clinical Mentorship Program (CMP)

In consultation with the Ethiopian Society of Obstetrics and Gynaecology (ESOG), Transform HDR has been implementing the CEMONC clinical mentorship in ten hospitals eligible for the program. Five of the ten hospitals selected for clinical mentorship were from Somali, three in Afar, one in Benishangul Gumuz, and one in Gambella regions (Supplementary Files 1 and 2). The mentorship program primarily targeted IESOs at the selected health facilities. The CMP activities comprise mentor selection and orientation, sensitization workshops, mentorship inception, and onsite mentorship (Supplementary File 3). Experienced obstetricians/gynecologists with substantial maternity, training, and leadership expertise were chosen to provide this clinical mentorship program. The CMP began with sensitization workshops in each of the four DRS following mentors' orientation. This is followed by mentorship program inception and subsequent visits. Need-based on-the-job training was also done at different times to build mentees' capacity and achieve a quality CEmONC service. One-on-one case management refers to accepting, assessing, diagnosing, treating, and following up on cases by the mentee together with a mentor. The mentors were also oriented on the use of case-scenario discussions on a problem using real-life constraints for the mentee to develop a capacity to anticipate how a specific situation might play out in the real world and to avoid potentially adverse outcomes rather than attempting to solve a problem that is easier to prevent. Furthermore, during the orientation, the importance of reviewing medical records, telephone mentoring, and organizing needs-based on-the-job training was elaborated to improve documentation and the mentee's capacity to perform and build confidence. During each visit, the mentor observed mentees using preset checklist while performing CS, demonstrating procedures to mentees, giving feedback, performing hands-on practice with the mentees, and holding feedback with health facility managers in the presence of a USAID Transform HDR representative. The CMP was conducted for 6 consecutive rounds with six days onsite by 11 senior obstetrics and gynecologists with substantial mentorship and leadership experiences (Supplementary File 4).

### Intervention component 2. Health facility support

Health facility support includes Comprehensive interventions to strengthen and complement the clinical mentorship program. The general health facility support provided for 15 hospitals consists of the provision of essential CEmONC equipment (e.g., purchase and equipment with mini-blood bank refrigerators), emergency blood transfusion service through establishing a mini blood bank, follow-up supervision, and establishment of Neonatal Intensive Care Unit (NICU) in hospitals and capacity building through provision of need-based training (e.g., on the clinical use of blood and blood products (ACUBBP) and post-ACUBBP training and follow-up supervision for laboratory experts, nurses, and physicians working in the designated institutions), and establishes a mini blood bank (Supplementary File 4).

### Measurements

#### Primary outcome

- Availability of CEmONC services: if the CEmONC signal function was functional in the three months prior to the survey, it was coded as “1” and otherwise “0”.

#### Secondary outcome

- CEmONC service use: measured in terms of the number of births, CS, and blood transfusion
- Obstetric complications: acute conditions arising from a direct cause of maternal death, such as antepartum or postpartum hemorrhage, obstructed labor, postpartum sepsis, complications of abortion, pre-eclampsia or eclampsia, ectopic pregnancy, and ruptured uterus, or indirect causes such as anemia [3] in the six months before USAID’s HDR intervention and 12 months following the intervention
- Institutional maternal mortality: the number of maternal deaths among total deliveries in the study hospitals in the six months prior to USAID’s HDR intervention and 12 months following the intervention.
- Institutional neonatal mortality: the number of neonatal deaths in the first 28 days of life among total deliveries in the selected hospitals in the previous six months before USAID’s HDR intervention and 12 months following the intervention
- C/S delivery: the number of CS delivery
- Obstetric referral for blood transfusion: the number of mothers referred from one health facility to the next higher or similar facilities for life-saving blood transfusion.

#### Operational definition

- CEmONC services:—are a set of key obstetrics services or signal functions for pregnant women and newborns who may experience fatal complications, such as severe bleeding, infection, prolonged or obstructed labor, eclampsia, and asphyxia in the newborn. The nine CEmONC signal functions consist of parenteral administration of antibiotics, treatments for eclampsia (provision of anticonvulsants), parenteral administration of oxytocin, assisted vaginal delivery (vacuum extraction), manual removal of placenta, removal of retained products of conception (MVA), newborn care, blood transfusion, and caesarian delivery of fetus in emergencies [11].

### Data collection

Twelve experienced data collectors (six midwives and six public health professionals) participated in the baseline survey. Six senior and experienced obstetrician-gynecologists were recruited to supervise the data collection process. Both data collectors and supervisors received a three-day training on collecting data. The training aimed to build a shared understanding of the contents of the data collecting tool, how to fill out each question, interviewing techniques, case selection, and field protocols to be followed during the survey so that the quality of data collection was achieved and ensured. Data was collected through face-to-face interviews with hospital administrators and maternity & newborn care service coordinators. Maternal and neonatal care records were also reviewed. All study hospital administrators and maternity & newborn care service coordinators were interviewed. In addition, the delivery, CS procedures, maternal admission and discharge logbooks, two neonates’ charts with breathing difficulties, two preterm deliveries with birth weights less than 2000 g, and two cesarean section operation notes per hospital were selected for chart review.

The data collection tool was adopted from an Adapted Averting Maternal Death and Disability tools (AMDD) [20]. The adapted data collection tool consists of structured questions to assess the facility’s infrastructure, CEmONC signal functions availability, Partograph Review; Caesarean Delivery recorded Review, and Newborn Complications Chart Reviews.

### Data analysis

Data were entered into CSPro 6.1 programming and exported to SPSS version 20.1 (IBM SPSS Statistics for Windows, Armonk, NY) for further analysis. We used descriptive statistical methods to summarize the relative number of CEmONC functions and others collected during baseline and post-intervention assessments. Lessons learned from USAID transform HDR post-intervention support activities were also described.

## Results

### Characteristics of the study hospitals

The baseline assessment covered 15 hospitals. The admission bed capacity of the hospitals ranges from 6 beds at Bullen Primary Hospital to 204 at Karamara General Hospital. The Obstetrics and Gynecology bed share varies from a single bed in Bullen to 40 beds in Karamara and Dubti. Two of the 15 hospitals were referral centers, six were general hospitals, and seven were primary hospitals where all hospitals are expected to provide all CEmONC signal function services (Supplementary File 1). Thirteen of the fifteen hospitals were fully operating, while the remaining two were partially operational with side expansions ongoing. All hospitals were supplied with electricity and water from various sources; they had separate rooms for delivery and PNC services. The majority of the hospitals lacked mini-blood banks, while some hospitals lacked separate rooms for ANC, post-CS, NICU, and newborn care (Table 1).

**Table 1:** Availability of necessary resources in the study Hospitals at baseline in DRS, 2019

Available resources		Number (%) of hospitals(*n* = 15)
Infrastructure		
Electricity supply	Grid	11(73.33)
	Generator	1(0.06)
	Solar	3(0.20)
	Backup generator	15(100.00)
Infrastructure Water supply	Pipeline	13(86.67)
	Well water	2 (13.33)
**Separate rooms for**		
ANC service		13(86.67)
delivery services		15(100.00)
PNC services		15(100.00)
post CS		8(53.33)
new born care		11 (73.33)
NICU		10(66.67)
Mini blood bank		3(0.20)

*ANC* Antenatal care, *PNC* Postnatal care, *NICU* Neonatal intensive care unit

### Availability of CEmONC services

**Fig. 1:**
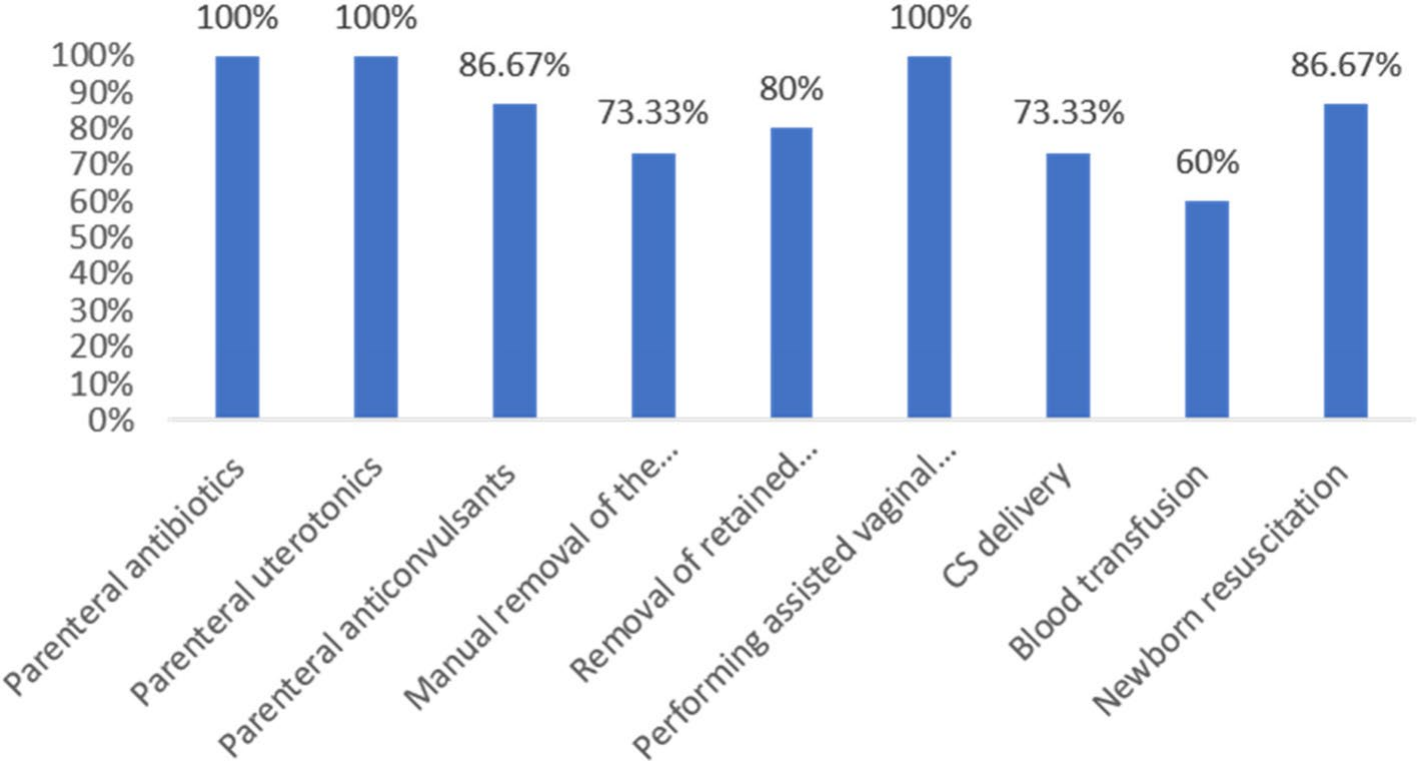
Performance of CEmONC signal functions at hospitals in DRS in Ethiopia (*n* = 15)

All 15 hospitals perform parenteral antibiotics, parenteral uterotonics, and assisted vaginal birth with Vacuum or forceps delivery. Blood transfusion was the least performed signal function in ten hospitals. CS service was performed in eleven hospitals, whereas it was not performed in Dalifage in Afar region, Bullen and Wanbara in Benishangul Gumuz region, and Pugnido in Gambella region due to lack of supplies, equipment, drugs, and trained personnel (Fig. 1).

#### CEmONC: Comprehensive Emergency Obstetric and Neonatal Care

**Fig. 2:**
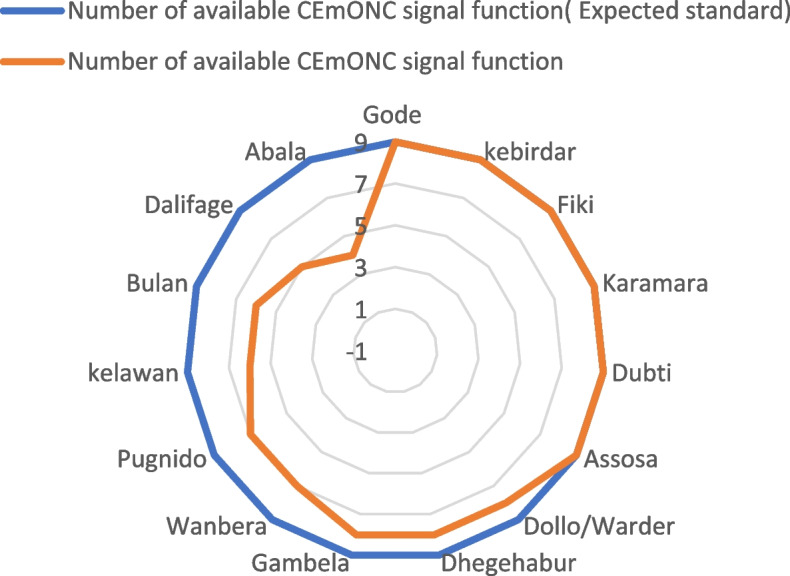
The availability of CEmONC signal functions in DR's hospitals, Ethiopia, 2019

Of 15 hospitals, less than half (i.e., Gode, Kebirdar, Fiki, Karamara, Dubti, and Asosa) provided all CEmONC signal functions, while the others partially performed the signal functions. Nine hospitals were not providing CS delivery or blood transfusion services or manual removal of placenta or parenteral anticonvulsants prior during the last three months before the baseline assessment. The blood transfusion signal function was unavailable in 6 of the 15 hospitals (i.e., Dhegaharbur, Abala, Kelawan, Bulan, Wanbara, and Pugnido hospitals). Caesarean Section delivery was not available in 4 hospitals (Dalifage, Bulan, Wanbara, and Pugnido), Manual removal of placenta service was not available in 4 hospitals (i.e., Abala, Kelawan, Bulan, and Gambella hospitals), and parenteral anticonvulsants signal function was not available in two hospitals (i.e., Dalifage and Abala) (Fig. 2).

#### Clinical mentorship program

Except for Abala, all hospitals selected for CEmONC clinical mentorship provide all nine CEmONC signal functions following the transform HDR-supported clinical mentorship program and comprehensive health facility support (Table 2). In addition, the mentees also reported that their clinical decision-making skills for cesarean section, documentation of anesthesia time, and completeness of operation notes using ultrasound at the labor ward, vacuum deliveries, interprofessional teamwork, and communication improved due to the skill transfer. Moreover, the use of WHO surgical safety checklists is routinized.

**Table 2:** Availability of the nine CEmONC signal functions in 10 hospitals of DRS in Ethiopia

Availability of CEmONC functions	Timing relative to the introduction of mentorship	
	Before October 2019 (*n* = 10)	After January 2021 (*n* = 10)
1. Parenteral antibiotics	10	10
2. Parenteral uterotonic	10	10
3. Parenteral anticonvulsants	8	10
4. Manual removal of placenta	8	10
5. Removal of retained products	7	10
6. Performing assisted vaginal delivery	10	10
7. CS delivery	9	9
8. Blood transfusion	8	10
9. Newborn resuscitation with bags	8	10

**Fig. 3:**
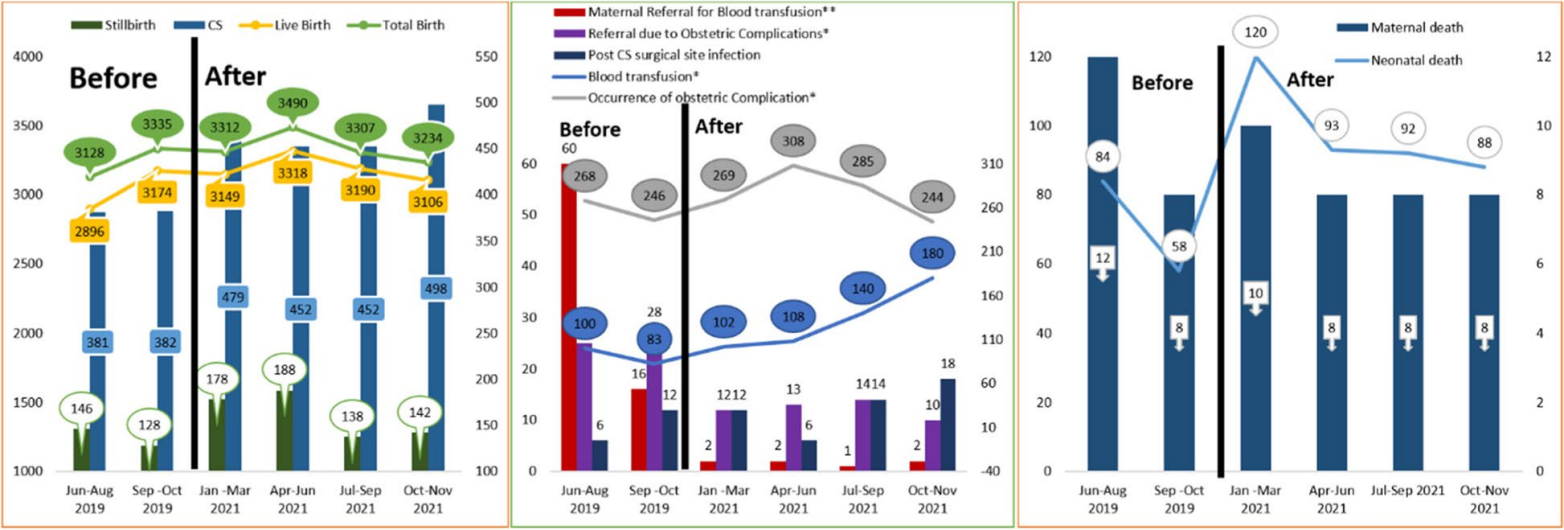
CEmONC service use, obstetric complication, Maternal and neonatal mortality before and after Transform HDR comprehensive Intervention, DRS, Ethiopia, 2021. *: The value indicates for eight hospitals; ** The value indicates for five hospitals

Regarding the quality of CEmONC signal functions services, there is no substantial change in CEmONC signal function service users, as evidenced by the quarterly total births and live births. However, following Transform HDR's comprehensive interventions, the number of Cesarean Sections increased by 7.25% at the last quarter of 2021compared to the third quarter of 20,219 and blood transfusion services has progressively increased. On the other hand, and the number of women referred for blood transfusions and further management of obstetric complications decreased by 96.67% at the last quarter of 2021 compared to the third quarter of 20,219. In contrast, there were fluctuations in the number of women with post-CS surgical site infection (SSI), obstetric complications, facility maternal deaths, deaths in the first 28 days of life, and stillbirths. The number of maternal deaths owing to preventable obstetric complications in hospitals in DRS varied by month and hospital (Fig. 3).

## Discussion

The study is the first to report the availability of CEmONC services in hospitals in the developing regional states in Ethiopia and to our knowledge there is no similar studies employing the same or similar methodologies to compare with the findings. The study highlighted the availability of CEmONC services before and after the Transform Health in Developing Region supported hospitals' performances regarding CEmONC signal function. However, the current study found that CEmONC signal function services before USAID Transform Health interventions in hospitals in the DRS in Ethiopia before the intervention are limited, although it varies by signal function and hospital type. Two-fifths of the hospitals perform all the nine CEmONC signal functions, and one-third of the CEmONC signal functions were performed at all hospitals. Blood transfusion, cesarean section (CS), manual placenta removal, removal of retained product of conceptus, and parenteral anticonvulsants were the least performed signal functions. For example, in eleven hospitals, CS delivery service is being provided, while blood transfusion services for needy mothers were supplied in ten hospitals. This means that hospitals in DRS would have likely passed up a chance to save the lives of women who would have died from preventable bad outcomes during pregnancy, delivery, or the immediate postpartum period if all CEmONC signal function services had been available.

The findings in the current study are in line with prior studies in Dire Dawa administrative city [14], Lubumbashi of the Democratic Republic of the Congo [15], Sindh region of Pakistan [16], and Kumba Health District of Cameroon [17] in which provision of EmONC signal functions vary by the health facility. In Dire Dawa administrative city in Ethiopia [14], blood transfusion, CS, manual removal of placenta, and removal of retained products are less performed signal functions that are consistent with the current study findings. Ntambue et al. [15] reported similar results that CEmONC signal function provision ranges from all to none between CEmONC facilities in Lubumbashi of the Democratic Republic of the Congo. In the Sindh region of Pakistan, even though BEmONC services were available in most CEmONC facilities, cesarean delivery and blood transfusion were unavailable [16]. The assessment of emergency obstetric and neonatal care services in Kumba Health District, Cameroon, from 2011 to 2014 found that neonatal resuscitation, manual evacuation of retained products, and the administration of anticonvulsants were the least likely to be performed [17]. This indicates that CEmONC signal functions are partially available in hospitals across developing countries. The availability varies from hospital to hospital, and more women are at risk of preventable cause of deaths.

All CEmONC signal functions were available in all ten hospitals after implementing CEmONC clinical mentorship and other support. This provides an insight that comprehensive support from relevant stakeholders could scale up the coverage of CEmONC signal functions services and its use at health facilities. The clinical mentorship program possibly improved clinical decision-making skills for cesarean section, anesthesia time documentation, operation notes' completeness, using ultrasound in the labor ward, vacuum deliveries, interprofessional teamwork, and communication due to skill transfer from mentors to mentees. The use of WHO surgical safety checklist is also routinized. However, despite a steady increase in the number of CS and blood transfusion services, the number of women referred for blood transfusions and further obstetric complication management has decreased as a result of the Transform HDR's comprehensive interventions, facility birth use, post-CS surgical site infection (SSI), obstetric complications, facility maternal deaths, deaths in the first 28 days of life, and stillbirths not changed. Also, the maternal mortality due to preventable obstetric complications in DRS hospitals is 2 to 3 times higher than the globally acceptable direct obstetric complications in emergency obstetric care facilities of less than 1% [21] following the comprehensive Transform HDR interventions. It indicates that CEmONC signal function availability alone does not rule out the possibility of maternal death even with CEmONC clinical mentorship. It signifies the need to identify factors that mainly contributed to the higher facility maternal death other than the unavailability of CEmONC signal functions in such context, which is crucial if it would have been accounted for in this study.

This is the first study to provide a local lens on the availability of CEmONC signal function and describe the importance of context-specific skill and experience transfer through a clinical mentorship program in a developing regional state in Ethiopia. However, this study is not without limitations. The quality of the underlying data sources may have influenced our findings; for example, our use of patient charts, logbooks, and administrative data as our data source there may have been biases introduced by overreporting, underreporting, omissions, and additions of information. The study did not consider situational and contextual factors like how COVID 19 and the ongoing conflict may have influenced the results. For example, experiences from 37 non-governmental organization-supported health facilities in low-income and middle-income countries show that COVID-19 disrupts the use of maternal health care [22]. The study showed the information generated from routinely collected data in Haiti, Lesotho, Liberia, Malawi, Mexico, and Sierra Leone resulted in significant drops in first antenatal care visits in Haiti (18%) and Sierra Leone (32%), as well as facility-based deliveries in all countries except Malawi between March and December 2020 [22]. Following COVID-19, movement restrictions and initially ambiguous safety measure direction seem to have impacted the overall mentorship program, resulting in a change in the visit and onsite skill transfer timetable.

Even if the findings fluctuate, the total number of facility births pre COVID-19 emergency in Ethiopia was maintained in the intervention hospitals, probably due to the Transform HDR-supported clinical mentorship program and the comprehensive health facility support from the USAID Transform HDR Activity. Moreover, the different time lengths considered to measure the CEMNOC service utilization before and after transform HDR interventions might have introduced a measurement bias.

One of the critical issues that have affected the finding is the disruption of services at Abala Hospital due to the ongoing threat of peace in northern Ethiopia. It is an important implication that Intercommunal conflicts often disrupt essential health service delivery and primarily affect women and children with downstream negative efforts to reduce morbidity and mortality. According to a regression analysis of data from 181 countries with armed conflict from 2000 to 2019, maternal mortality climbed by 36.9% per 100,000 live births, and infant mortality increased by 2.8 per 1,000 live births [23]. Hence, it would have been better if the study had investigated how the ongoing inter-communal conflicts affected the current study results particularly in the case of Abala hospital. It was beyond the capacity of researchers to anticipate the timing of the conflict and its expansion.

Furthermore, even if CEmONC signal functional are widely available at Transform HDR-supported hospitals, it is worth investigating which women are using them and what reasons drive high facility maternal and neonatal death. For example, distance to the facility, religiosity, socioeconomic class (e.g., education, literacy, income levels), and the facility's reputation all significantly impact maternal health service utilization [24, 25]. In addition, our study does not.

## Conclusion

The availability of CEmONC signal function services in the DRS in Ethiopia is limited and varies by type of signal function and hospitals that provide clinical services. Two-fifths of the hospitals perform all the nine CEmONC signal functions, and one-third of the CEmONC signal functions were performed at all hospitals. The least performed signal functions were blood transfusion, cesarean section (CS), manual placenta removal, removal of retained product of conceptus, and parenteral anticonvulsants. Lessons learned are that comprehensive support from relevant stakeholders could increase the availability of CEmONC signal function services at health facilities. However, the facility birth use, post-CS surgical site infection (SSI), obstetric complications, facility-based maternal deaths, neonatal death, and stillbirths not changed. This indicates the need for investigating underlying and proximal factors that contributed to maternal death and stillbirth in the Developing Regional State of Ethiopia. In addition, there is also the need to assess the quality of the CEmONC services in the supported hospitals, institutionalize reviews, surveillance, and response mechanism for maternal and perinatal or neonatal deaths and near misses.

## Supplementary Information

**Additional file 1.**

**Additional file 2.**

**Additional file 3.**

**Additional file 4.**

## Data Availability

All of the data used in this paper can be obtained by contacting Amref Health Africa at Derbe.Tadesse@amref.org.

## Abbreviations

AMDD: Averting Maternal Death and Disability tools
CEmONC: Comprehensive Emergency Obstetric and Neonatal Care
CMP: Clinical Mentorship Program
CS: Caesarian Section
DRS: Developing Regional States
EmONC: Emergency Obstetric and Neonatal Care
ESOG: Ethiopian Society of Obstetrics and Gynaecology
HDR: Health in Developing Region
LLMI: Low and Lower-Middle Income countries
SDGs: Sustainable Development Goals
SSA: Sub-Saharan African
