# Supplementary material for: Availability of comprehensive emergency obstetric and neonatal care in developing regions in Ethiopia: lessons learned from the USAID transform health activity
Source: BMC Health Serv Res. 2022 Nov 2;22:1307. doi: 10.1186/s12913-022-08712-w (PMC9628556; doi:10.1186/s12913-022-08712-w)
Supplement: Supplementary file 1 — Additional file 1. [file 12913_2022_8712_MOESM1_ESM.docx]

**Supplementary File 1. Characteristics of the selected hospitals in DRS**

| Facilities | Region | Established in | Catchment population | Number of beds | OB &GYN |
| --- | --- | --- | --- | --- | --- |
| 1. Warder Hospital | Somali | 2005 | 441,517 | 86 | 6 |
| 1. Gode Hospital | Somali | 1952 | 1,000,000 | 13 | 13 |
| 1. Kebridahar Hospital | Somali | 1948 | 280,0 | 129 | 15 |
| 1. Fik Hospital | Somali | 2003 | 550,000 | 82 | 17 |
| 1. Dhegahbur Hospital | Somali | 1983 | 633,004 | 122 | 36 |
| 1. Karamara Hospital | Somali | 1950 | 1,282,533 | 204 | 40 |
| 1. Dubti Hospital | Afar | 1966 | 1,000,000 | 140 | 40 |
| 1. Ab’ala Primary Hospital | Afar | 2015 | 61,500 | 25 | 9 |
| 1. Dalifage Hospital | Afar | 2009 | 76,000 | 40 | 10 |
| 1. Kelewan Hospital | Afar | 2010 | 292000 | 40 | 9 |
| 1. Assosa General Hospital | Benishangul Gumuz | 1984 | 650,000 | 120 | 30 |
| 1. Wonbera Primary Hospital | Benishangul Gumuz | 2018 | 84,876 | 10 | 5 |
| 1. Bullen Primary hospital | Benishangul Gumuz | 2018 | 64,390 | 6 | 1 |
| 1. Gambella Hospital | Gambella | 1995 | 468,017 | 141 | 16 |
| 1. Pugnido Hospital | Gambella | 2009 | 86,416 | 35 | 12 |
