# Supplementary figures and images for: Availability of comprehensive emergency obstetric and neonatal care in developing regions in Ethiopia: lessons learned from the USAID transform health activity
Source: BMC Health Serv Res. 2022 Nov 2;22:1307. doi: 10.1186/s12913-022-08712-w (PMC9628556; doi:10.1186/s12913-022-08712-w)

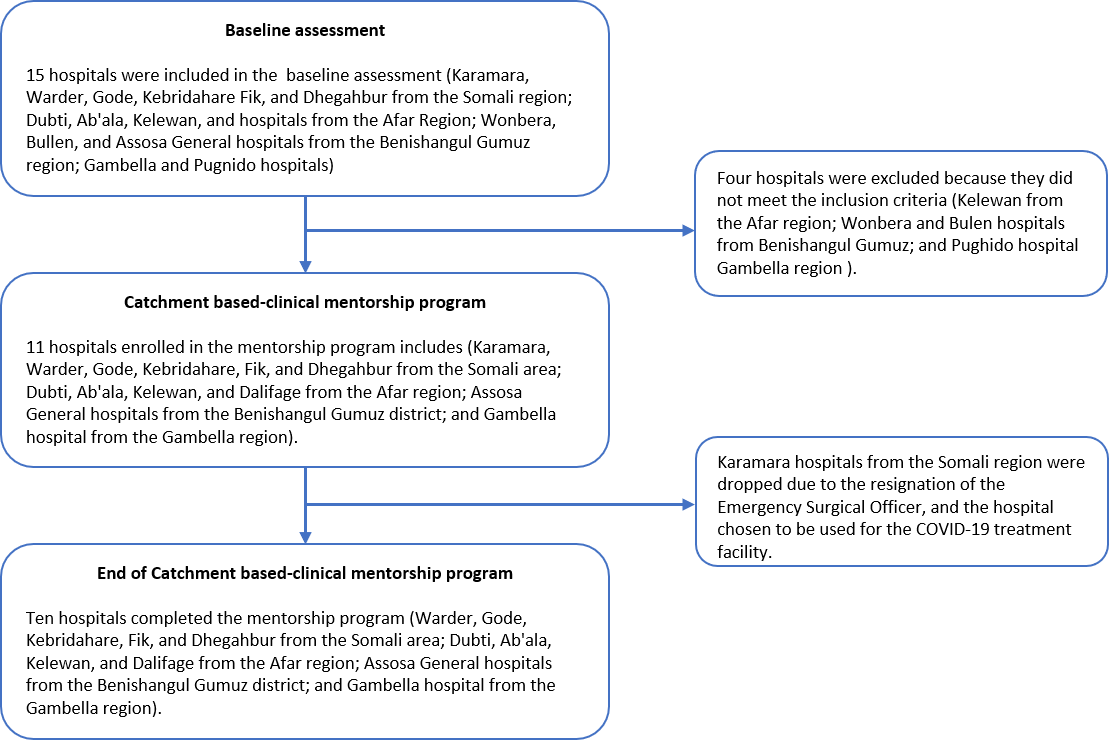


**Supplementary File2: Enrollment of DRS Hospitals in a CEmONC clinical mentorship program**

Supplement: Supplementary file 2 — Additional file 2. [file 12913_2022_8712_MOESM2_ESM.docx]

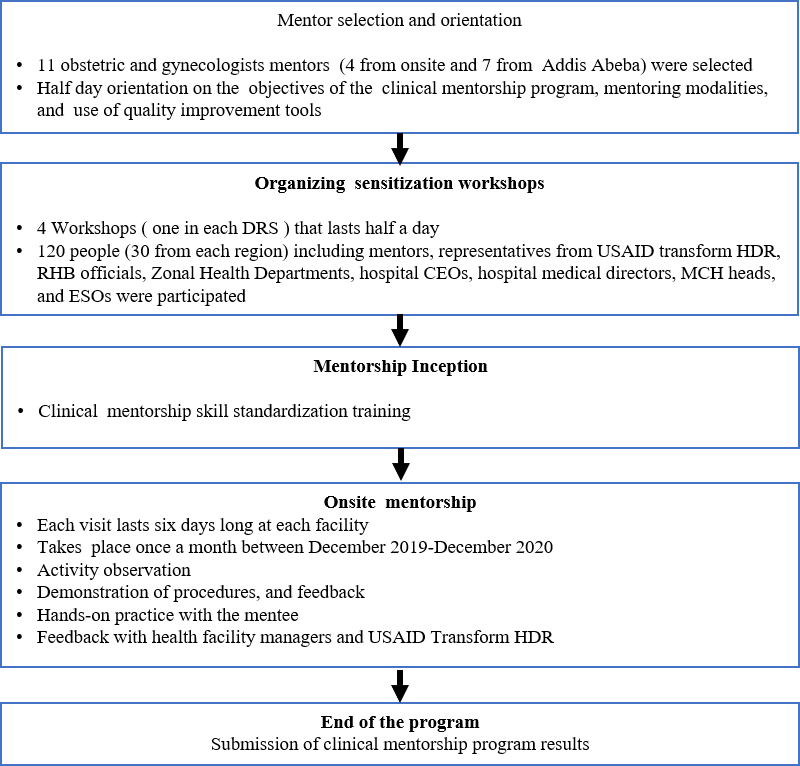


**Supplementary File 3: CEmONC clinical mentorship program activities in DRS hospitals**

Supplement: Supplementary file 3 — Additional file 3. [file 12913_2022_8712_MOESM3_ESM.docx]
