# Supplementary material for: Availability of comprehensive emergency obstetric and neonatal care in developing regions in Ethiopia: lessons learned from the USAID transform health activity
Source: BMC Health Serv Res. 2022 Nov 2;22:1307. doi: 10.1186/s12913-022-08712-w (PMC9628556; doi:10.1186/s12913-022-08712-w)
Supplement: Supplementary file 4 — Additional file 4. [file 12913_2022_8712_MOESM4_ESM.docx]

**Supplementary File 4. USAID funded CEmONC interventions**

| **Intervention components** | **Purpose** | **Features/characteristics of the interventions** |
| --- | --- | --- |
| Clinical Mentorship Program in ten selected hospitals: | To increase the availability and improve the quality of CEmONC signal functions through boosting ESOs clinical knowledge, skills, and attitudes at selected health facilities | Conducted for 6 consecutive rounds with six days onsite by 11 senior obstetrics and gynecologists with substantial mentorship and leadership experiences. The mentors had four days of clinical mentorship skill standardization training. The trainers were clinical mentorship experts from MOH, Ethiopia |
| Health facility support | To enhance CEmONC service availability in the selected health facilities through establishing mini blood banks and enhancing health care providers capacity for maternal and neonatal care. | Through the Health facility support program transform HDR:   1. Established -----Mini blood bank services through procurement of blood bank refrigerators 2. Supported Dubti, Assosa, Wonbera, Bulen, Gambella, Pugnido, Kebridahare, and warder Hospitals NICU with radiant warmer, phototherapy and neonatal incubators, medical equipment maintenance handling 3. Conducted capacity building trainings on  - appropriate clinical use of blood and blood products (ACUBBP) for…… laboratory experts, nurses, and physicians - medical equipment maintenance and handling … - Essential Care for Every Baby (ECEB), Essential Care for Small Baby (ECSB) and Helping Baby Breath (HBB) and Help Baby Survive (HBS) and Neonatal Intensive Care Unit (NICU) for a total of 420 health care professionals  1. Conducted post training monthly/quarterly follow up supervision 2. Distribution of ENC/ECSB/HBB treatment flow chart and algorithm to all hospitals |
